# De novo whole-genome assembly and annotation of *Coffea arabica* var. Geisha, a high-quality coffee variety from the primary origin of coffee

**DOI:** 10.1093/g3journal/jkae262

**Published:** 2024-11-15

**Authors:** Juan F Medrano, Dario Cantu, Andrea Minio, Christian Dreischer, Theodore Gibbons, Jason Chin, Shiyu Chen, Allen Van Deynze, Amanda M Hulse-Kemp

**Affiliations:** Department of Animal Science, University of California Davis, Davis, CA 95616, USA; Department of Viticulture and Enology, University of California Davis, Davis, CA 95616, USA; Genome Center, University of California Davis, Davis, CA 95616, USA; Department of Viticulture and Enology, University of California Davis, Davis, CA 95616, USA; Computomics GmbH, 72072 Tübingen, Germany; Computomics GmbH, 72072 Tübingen, Germany; Pacific Biosciences, Menlo Park, CA 94025, USA; Department of Plant Sciences, University of California Davis, Davis, CA 95616, USA; Department of Plant Sciences, University of California Davis, Davis, CA 95616, USA; Department of Plant Sciences, University of California Davis, Davis, CA 95616, USA

**Keywords:** Geisha coffee, *Arabica* coffee, hybrid genome assembly, high-quality coffee, coffee center of origin

## Abstract

Geisha coffee is recognized for its unique aromas and flavors and, accordingly, has achieved the highest prices in the specialty coffee markets. We report the development of a chromosome-level, well-annotated, genome assembly of *Coffea arabica* var. Geisha. Geisha is considered an Ethiopian landrace that represents germplasm from the Ethiopian center of origin of coffee. We used a hybrid de novo assembly approach combining 2 long-read single molecule sequencing technologies, Oxford Nanopore and Pacific Biosciences, together with scaffolding with Hi-C libraries. The final assembly is 1.03 Gb in size with BUSCO assessment of the assembly completeness of 97.7% of single-copy orthologs clusters. RNA-Seq and Iso-Seq data were used as transcriptional experimental evidence for annotation and gene prediction revealing the presence of 47,062 gene loci encompassing 53,273 protein-coding transcripts. Comparison of the assembly to the progenitor subgenomes separated the set of chromosome sequences inherited from *Coffea canephora* from those of *Coffea eugenioides.* Corresponding orthologs between the 2 *Arabica* varieties, Geisha and Red Bourbon, had a 99.67% median identity, higher than what we observe with the progenitor assemblies (median 97.28%). Both Geisha and Red Bourbon contain a recombination event on chromosome 10 relative to the 2 progenitors that must have happened before the geographical separation of the 2 varieties, consistent with a single allopolyploidization event giving rise to *C. arabica.* Broadening the availability of high-quality genome assemblies of *C. arabica* varieties paves the way for understanding the evolution and domestication of coffee, as well as the genetic basis and environmental interactions of why a variety like Geisha is capable of producing beans with such exceptional and unique high quality.

## Introduction

Coffee is one of the most popular beverages worldwide, with an estimated consumption of 400 billion cups per year (Sachs *et al*. 2019). While there are over 124 *Coffea* species (Davis *et al*. 2011), the bulk of the coffee consumption comes from 2 species, *Coffea canephora* (Robusta) and *Coffea arabica* (*Arabica*). *Arabica* represents 58% of world coffee production and the remaining comes from Robusta (ICO 2023). *Arabica* coffee has been the preferred coffee because of its refined taste, with a rich and well-balanced flavor profile (DaMatta *et al*. 2007).

**Fig. 1.**
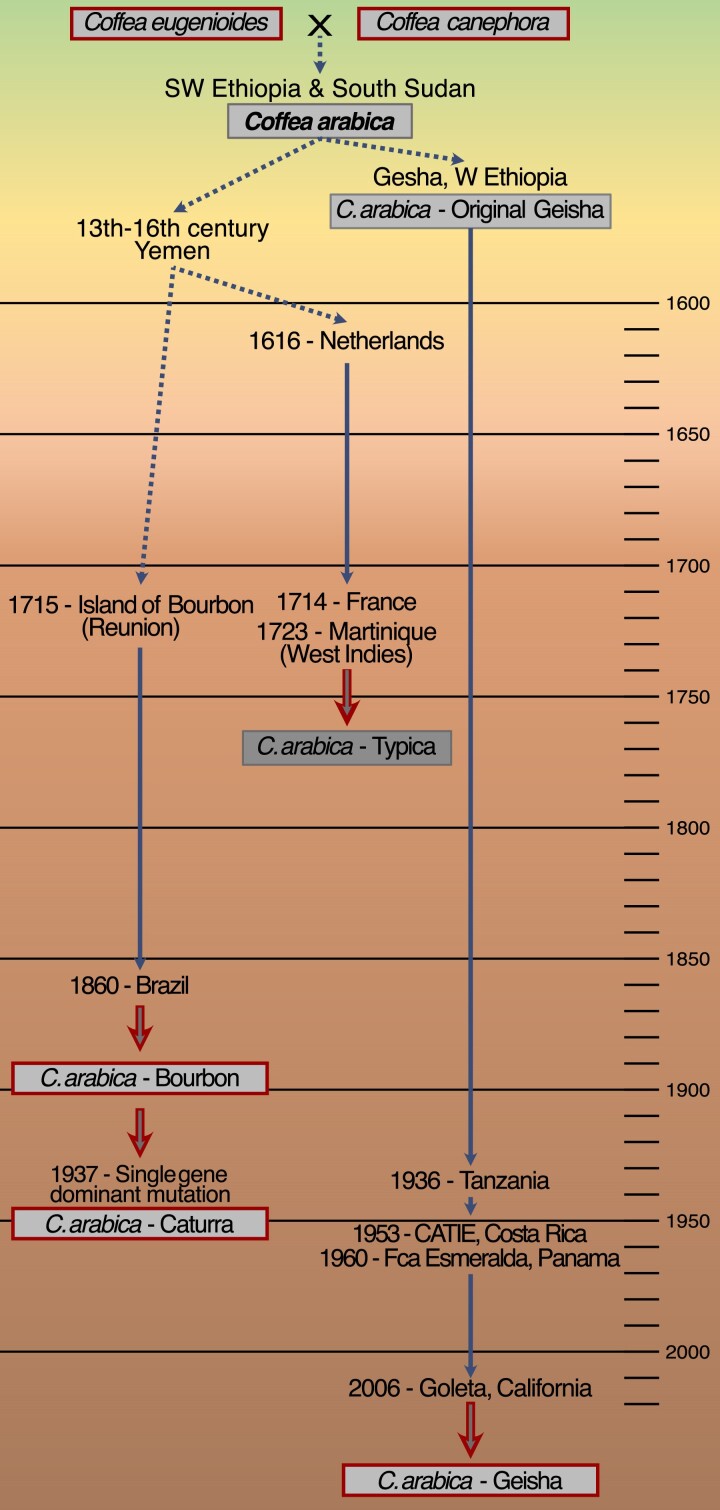
Time-line of origin and migrations of *C. arabica* varieties: *C. arabica* originated from the hybridization of the 2 diploid progenitor species *C. eugenioides* and *C. canephora* and a single allopolyploidization event. From the center of origin, one *C. arabica* variety, Bourbon, was derived from the early migration of *C. arabica* germplasm through Yemen*. C. caturra* later was derived from Bourbon by a single mutation event*. C. arabica*, Geisha, on the other hand, has a distinct direct connection to the center of origin of coffee and is considered a *C. arabica* landrace, as it travelled only in recent times from Ethiopia to California. Species and varieties for which a genome assembly is available are highlighted with red boxes. CATIE, Tropical Agricultural Research and Higher Education Center.

*C. arabica* has an allotetraploid genome with *n* = 22 chromosomes derived from the hybridization of its maternal diploid progenitor species *C. eugenioides* (E genome) and its paternal progenitor, *C. canephora* (C genome), or Robusta coffee, from a single allopolyploidization event about 100,000 years ago (Lashermes *et al*. 2016). In contrast to the other *Coffea* species, *C. arabica* is the only species that is largely autopollinated, with ∼10% natural cross-pollination (Carvalho 1985). The center of origin and diversity of *C. arabica* is considered to be southwestern Ethiopia and southern Sudan (Lashermes *et al*. 2016; Montagnon *et al*. 2021). The original germplasm of the cultivated *Arabica* varieties outside Ethiopia transited from the center of origin to Yemen (Montagnon *et al*. 2021) from where it spread worldwide, giving origin to 2 similar germplasms, Typica and Bourbon, that later arrived in the Americas in the 17th and 18th centuries (Scalabrin *et al.* 2020). Currently, 2 high-quality coffee genome assemblies have been produced, IGA-CARA 2.4 from the Red Bourbon variety (Scalabrin *et al*. 2024) and CARA 1.0 (unpublished; NCBI RefSeq assembly GCF_003713225.1) from the variety Caturra that is derived from a single dominant mutation from Bourbon (Krug *et al*. 1949). Assemblies of the 2 progenitor species, *C. canephora* (Denoeud *et al*. 2014) and *C. eugenioides* (NC_040035.1), are also available. Figure 1 shows a brief summary of the geographical origins of the current *C. arabica* varieties emphasizing the unique origins of the Geisha variety.

We developed a chromosome-level, well-annotated, genome assembly of *C. arabica* var. Geisha, considered an Ethiopian landrace, representing germplasm from the Ethiopian center of origin of coffee. The Geisha variety originated in the forests of the Gesha region in Western Ethiopia (Fig. 1) and was collected around 1936 by Captain Richard Whalley, a British Consul for the Bench Maji region. Seeds were first sent to Kenya and then to the Lyamungu Research Station in Tanzania. From there, seeds were brought in 1953 to the Center for Tropical Agricultural Research and Education (CATIE) in Costa Rica (Boot 2013). In the mid-1960s, Geisha was planted from the CATIE germplasm in a small number of coffee farms in Boquete, Panama (Price Peterson, personal communication; Boot 2013). DNA analysis confirmed the origin of the CATIE Geisha as the original Geisha tree preserved in Tanzania (WCR 2016). Additionally, a study comparing Ethiopian forest Geisha-type samples with Panamanian Geisha plants confirmed the high likelihood of the origin of the Panamanian Geisha from the Ethiopian forest (Krishnan and Boot 2014). Geisha coffee gained prominence in 2005 for its unique, excellent quality when Hacienda Esmeralda, owned by the Peterson Family in Boquete, Panama, won the “Best of Panama Competition,” as well as setting a world record for the highest auction price per pound of coffee at the time (WCR 2016). In 2006, a handful of Geisha coffee seeds were sent from Panama by Price Peterson to Jay Ruskey at Goodland Organics in Goleta, California. This introduction laid the foundation for the Geisha variety plants currently established at Goodland Organics and in Southern California, from where we collected plant material to develop our chromosome-level genome assembly (UCD v.1.0).

In this study, we utilized long-read sequencing based on Oxford Nanopore Technologies (ONT, Oxford, England) and Pacific Biosciences (PB, Menlo Park, USA), together with scaffolding with Dovetail (Scotts Valley, USA) genome-wide chromatin conformation capture technologies and used transcript evidence, to develop a chromosome-level, well-annotated genome assembly of *C. arabica* variety Geisha. This paves the way for understanding the evolution and domestication of coffee, as well as the genetic basis of why a variety like Geisha is capable of producing beans with such exceptional and unique high quality and how environmental interactions like altitude and changing climate parameters can affect quality and yield in coffee.

## Materials and methods

### Plant material collection and extraction of nucleic acid

Young leaf tissues were collected from coffee plant UCG-17 of the variety “Geisha” (*C. arabica*) at Goodland Organics in Goleta, CA. For long-read sequencing, leaves from the second youngest node were processed using a modified CTAB extraction protocol as described in Stoffel *et al*. (2012) to produce high molecular weight (HMW) DNA. Second node leaves were also utilized for DNA extraction using the Qiagen DNeasy Plant Kit for short-read sequencing. DNA concentration was measured using QuBit (Thermo Fisher Scientific, Waltham, USA), and fragment size and integrity were evaluated with an Agilent 2100 Bioanalyzer (Agilent Technologies, Inc., Santa Clara, USA).

For transcriptome sequencing, we collected samples of different organs and tissue types in RNAlater (Thermo Fisher Scientific, Waltham, USA) at different developmental stages (Supplementary Table 1), from Geisha plants at the same Goleta, CA location. For RNA-Seq, each tissue was extracted using Qiagen RNeasy Plant Kits. For Iso-Seq, RNA was extracted using the CTAB method as reported in Blanco-Ulate *et al*. (2013). The integrity of RNA samples was evaluated on an Agilent 2100 Bioanalyzer (Agilent Technologies, LLC).

### Genome size estimation with flow cytometry

For genome size estimation, a ∼50-mg sample of coffee leaves from the Geisha plant UCG-17 was collected in Goleta, California and shipped between moist paper towels on cooling packs for estimation of genome size using flow cytometry. Intact nucleus preparation was performed at Benaroya Research Institute, Seattle, WA, and nuclear content was estimated by taking an average of 4 repeated estimates. The resulting estimate of genome size was 2.22 ± 0.030 pg/2C or 1.09-Gb haploid genome size.

### Genomic library preparation and sequencing

A whole-genome sequencing Illumina library with an average insert size of 350 bp was sequenced on the Illumina HiSeq X machine producing a total of 37× genome coverage with 150-bp paired-end reads. Quality of sequencing was assessed with FASTQC software v.0.11.2. Reads were processed to remove adapters and trim low-quality bases using CLC Genomics Workbench (Qiagen, Valencia, USA).

PB SMRT sequencing libraries were constructed according to the manufacturer’s recommendations at the UC Davis Genome Center. Fragments > 10 kb were size selected for sequencing via BluePippin (Sage Science, LLC). The library was sequenced using 101 SMRT cells of the P6-C4 technology on the RS II. A total of 95.3 Gb of sequence was produced equivalent to 79.4× genome coverage. N50 of reads ranged from 16 to 21 kb. Cleaned reads were utilized for genome assembly with FALCON-UNZIP v.0.4.0 followed by 2 rounds of Quiver for base correction, to obtain primary contigs and secondary haplotigs. A final round of polishing was performed using PILON software v.1.23 (Walker *et al*. 2014) and whole-genome sequencing short-read data to correct for SNPs and small indels.

An ONT library was constructed with 250-kb averaged size DNA fragments according to the manufacturer’s recommendations at the UC Davis Genome Center. The library was sequenced on 2 flow cells on a Promethion (ONT), which generated 150× genome coverage. The CANU v.1.8 software (Koren *et al*. 2017) was used to produce an assembly with the ONT sequence. That assembly was polished with the ONT raw reads using Racon software v.1.3.3 (Vaser *et al*. 2017), resulting in ONT assembly v.0.5.

Chicago and Hi-C libraries (Dovetail) were produced from HMW DNA. The Chicago library was sequenced and produced 115.0 Gb of data, corresponding to a depth of genome coverage of 88.5× X-Fold. Hi-C library sequencing produced 84.5 Gb of reads, corresponding to 65× X-Fold coverage. The standardized Dovetail informatics pipeline (HiRise) was utilized with the FALCON-UNZIP polished primary contigs fasta files as input. Briefly, the pipeline first processes the Chicago sequencing data and corrects short-range distance information and then utilizes the Hi-C sequencing information for further scaffolding of long-range information. A number of breaks/joins were investigated manually to confirm the software functionality. Plots showing the resulting Hi-C linkages were also investigated manually. This process utilizing the Chicago and Hi-C libraries was repeated utilizing the v.0.5 ONT assembly after which the scaffolded version of the ONT assembly was polished with an additional round of Racon v.1.3.3 (Vaser *et al*. 2017) using the PB raw reads to produce the v.1.0 ONT Dovetail assembly. Plots showing the resulting Hi-C linkages were investigated manually. Standard assembly quality statistics were evaluated for each assembly version, including gaps vs number contigs and scaffolds.

### Assembly comparisons and pseudomolecule generation

The PB Dovetail assembly was aligned to the v.1.0 ONT Dovetail assembly using RaGOO v.1.1 (Alonge *et al*. 2019). Scaffolds in the PB Dovetail assembly were further scaffolded according to the alignment with the v.1.0 ONT Dovetail assembly to produce a final draft assembly. This final draft assembly was aligned with MUMmer software v.3.0 (nucmer algorithm) (Kurtz *et al*. 2004) to determine relationships with the progenitor genome assemblies available at NCBI—*C. canephora* (GCA_900059795.1) and *C. eugenioides* (GCA_003713205.1). Global alignments were visualized with minimum lengths of 5,000 bases and both 98 and 99% identity between the sequences. Pseudomolecule pair alignments that dropped off by increasing the percent identity to 99% represent the scaffold corresponding to the opposite subgenome homeolog. The correlation of this identity was used to assign and name the pseudomolecules to each subgenome. Chromosome pairs were numbered following the *C. canephora* genome (Dereeper et al. 2013), with “c” or “e” indicating the subgenome of inheritance from *C. canephora* or *C. eugenioides*, respectively. For example, Chr01e refers to ortholog to chromosome 1 of the *C. eugenioides* genome while Chr01c refers to ortholog of chromosome 1 of the *C. canephora* genome. The resulting assembly arranged in named pseudomolecules or chromosomes is designated the final version as coffee (*C. arabica*) genome UCD v.1.0 for variety Geisha. The completeness of the final genome assembly was assessed by searching highly conserved single-copy orthologs with BUSCO v.5.1 (Simao *et al*. 2015) using Eudicots odb10 database of gene models.

### Genome annotation

Repetitive sequences were identified in the final draft assembly using RepeatModeler 2.0 (Smit and Hubley 2019), to identify repeat families present specifically in the coffee genome. A custom coffee repeat library was produced and then used as input with the final genome assembly with RepeatMasker v.4.1.0 (Smit *et al*. 2013) to identify repeat positions in the genome and to generate a masked version of the genome sequence. This masked version was used as the input for the annotation procedure that followed.

High-quality Iso-Seq data were used to produce high-quality gene models for training gene predictors in PASA v.2.4.1 (Haas 2003) along with transcript evidence obtained from RNA-Seq data aligned on the genome using HISAT2 v.2.1.0 (Kim *et al*. 2015) and by performing transcriptome assemblies using Stringtie v.2.0 (Pertea *et al*. 2015) and Trinity v.2.8.5 (Grabherr *et al*. 2011). Public databases (Swiss-Prot and UniProt Nov. 11, 2020), transcriptome assemblies, and the Iso-Seq data described above were used as transcript and protein evidence. They were mapped on the genome using PASA v.2.4.1 (Haas 2003) and Exonerate v.2.4.0 (Slater and Birney 2005). Ab initio predictions were generated using Augustus v.3.3.3 (Stanke *et al*. 2006), GeneMark v.4 (Lomsadze 2005), and GlimmerHMM v.3.0.4. EvidenceModeler v.1.1.1 (Haas *et al*. 2008), and PASA used these predictions to generate consensus gene models. Models showing untranslated regions or introns longer than 25 kb were removed, as well as models encoding for proteins with no ortholog correspondence (50% of identity and coverage) with other known plant proteins in the RefSeq Database (January 17, 2017). The final functional annotation was produced by combining Diamond v.2.0.4 (Buchfink *et al*. 2014) hits against the UniProt protein database and InterProScan v.5.40-77.0 (Jones *et al*. 2014) outputs using Blast2GO v.4.1.9 (Gotz *et al*. 2008).

### Comparison of genome assembly and synteny analysis

Putative pseudogenes in *C. arabica* var. Geisha genome were identified by reciprocal mapping of the annotated coding sequences (CDS) using BLAT v.36 × 2 (Kent 2002). Alignments with an identity > 50% and a coverage of 80% of the CDS were considered to allow flexibility in terms of sequence identity while ensuring the match would encompass significantly conserved genomic regions. Only those matches not overlapping any other annotated gene locus and encoding for an incomplete ORF (e.g. premature stop codon and lack of methionine at start) were considered putative pseudogenes.

Relationships between ortholog protein-coding genes of the 2 subgenomes and the parental species *C. canephora* (NCBI assembly and annotation GCA_900059795.1) and *C. eugenioides* (NCBI assembly and annotation GCF_003713205.1) were established searching for homolog proteins with BLASTp v.2.7.1+ (Altschul *et al*. 1990), using a minimum *e*-value 0.001 and collecting only the best 100 hits. Synteny between the progenitor species with the corresponding subgenomes and between the 2 subgenomes was performed with MCScanX v.11.Nov.2013 (Wang *et al*. 2012). To relate homologous loci across different genomes, coding genes were associated using the hits from protein BLAST searches; pseudogenes were associated with annotated coding genes. Syntenic blocks were defined with a minimum of 15 genes per block and a maximum stretch of 10 genes missing per block.

The assembly structure of *C. arabica* variety Geisha was compared with the assembly of *C. arabica* variety Red Bourbon from Scalabrin *et al*. (2024) (IGA_Cara_2.4, NCBI accession GCA_030873655.1). Whole-genome sequence alignments were produced between each pair of genomes and subgenomes using Minimap2 v.2.26-r1175 (parameters: “-k19 -w19 -U50,500 -A1 -O6,26 -s200”) (Li 2018). To compare the sequence identity across multiple assemblies, *C. arabica* variety Geisha and *C. arabica* variety Red Bourbon genome sequences were sliced into 25-kb windows using makewindows (parameters: “-w 25000”) and getfasta tools from the BEDtools suite v.2.24.0 (Quinlan and Hall 2010). The sequences of the 25-kb windows were then mapped against all other subgenomes pseudomolecules and progenitor genome assemblies (*C. canephora* and *C. eugenioides*) using Minimap2 v.2.26-r1175 (parameters: “-x map-hifi”) (Li 2018). The coverage and identity for each hit were calculated. For each genomic window in each comparison, the hit showing the highest number of matching nucleotides was selected as the best one.

## Results and discussion

### *C. arabica* genome size estimates

*Coffea* species have been estimated to have a wide range of genome sizes, from the smallest reported diploid genome of 0.95 ± 0.13 pg in *C. racemosa* (Cros *et al*. 1995) to the largest in the genus ascribed to the *C. arabica*. This is consistent with *C. arabica* being an allotetraploid and, thus, having inherited 2 subgenomes by a hybridization event from 2 diploid progenitors, *C. canephora* and *C. eugenioides*. Both of the diploid progenitors of *C. arabica* appear to have fairly consistent genome size estimates using flow cytometry. *C. eugenioides* has a reported 2C of ∼1.36 pg and *C. canephora* a slightly larger 2C of ∼1.46 pg (Supplementary Table 2). However, the estimates for *C. arabica* not only have been reported to be more variable (Supplementary Table 2) than the progenitors, but also to be consistently smaller than their combined size of ∼2.82 pg.

Our flow cytometry analysis of *C. arabica* variety Geisha genome size reported a 2C value of 2.22 ± 0.030 pg. This estimate is marginally smaller than estimates previously reported for other *C. arabica* varieties (2.30–2.72 pg) but within the range of variability previously observed due to the occurrence of “intraspecies polymorphism,” the estimating technique used and the application of the methods to a tetraploid genome (Cros *et al*. 1994; Clarindo *et al*. 2013). From our flow cytometry estimate, the 1C nuclear genome size of *C. arabica* var. Geisha should ∼1.085 Gb (assuming 1 pg = 978 Mb; Dolezel *et al*. 2003).

### Assembly of Geisha genome

The genome of *C. arabica* variety Geisha (UCD v.1.0) was reconstructed using a hybrid de novo assembly approach combining 2 long-read single molecule sequencing technologies (PB SMRT technology and Oxford Nanopore ONT technology), together with Dovetail proximity-ligation sequencing technologies for scaffolding both assemblies (Table 1; Supplementary Fig. 1).

**Table 1.** Genome assembly and annotation statistics.

Assembly	Geisha -UCD1.0
Assembly length (kb)	1,025,607
Chromosomes	22
Unplaced sequences	214
%GC	37
Number of gaps	1,876
Total gap length (kb)	223.6
Median scaffold (kb)	134.9
Maximum scaffold (kb)	69,315.2
Scaffold N50 (kb)	43,715.0
Scaffold L50 (#)	10

	ONT based	PacBio Based
Scaffold N50 (Mb)	44.49	42.54
Scaffold L50 (#)	12	10
Total contigs	2,112	
Contig N50 (kb)	1,107.8	
Contig L50 (#)	234	
Annotation		
Number of gene loci	47,062 (8,617 pseudogenes)	
Number of proteins	53,273	
Number of complete BUSCO	2,272	
Number of missing BUSCO	30	
Complete BUSCO (%)	97.7	
Repeat (%)	60.6	

V5.1, Eudicots odb10 database, 2,326 BUSCOs.

Long-read data sets were first assembled separately. PB SMRT sequencing produced ∼87.8× coverage based on the 1.09-Gb genome size estimated by flow cytometry. PB long reads were assembled using the FALCON-Unzip pipeline (Chin *et al*. 2016) producing an assembly containing 1,316 primary contigs comprising 1.025-Gb total bases (94.42% estimated size) with N50 of 1.85 Mb. The primary assembly size suggested the assembly reported a nonredundant representation of 1 single haplotype for both subgenomes C and E.

ONT long-read sequencing generated ∼150×-fold coverage of the genome and was assembled using CANU v.1.8 (Koren *et al*. 2017), which produced 1,685 contigs comprising 1.18 Gb (109% estimated size) with N50 of 4.10 Mb. The CANU assembly procedure, unlike FALCON-Unzip, is not haplotype aware, and the increased assembly size with respect to the estimated flow cytometry can be attributed to the lack of phasing and compressing of the alternative alleles and causing the copresence of alternative haplotype sequences.

After polishing, both sets of assembled contigs underwent 2-stage scaffolding using 2 different Dovetail Genomics proximity-ligation sequencing technologies. The first scaffolding stage involved the use of a Chicago library, and the second stage involved a Hi-C library. Comparing the overall assembly statistics after scaffolding of the PB and ONT assemblies (Table 1) showed that the ONT assembly was ∼1.04 times more contiguous based on scaffold N50 of 44.48 Mb in ONT vs 42.54 Mb in PB.

Initial BUSCO (Simao *et al*. 2015) (V3 with Eudicots odb9 database with 2,121 gene models) assessment showed that the PB assembly located 2,058 (97.1%) complete genes while the unpolished ONT assembly resulted in 1,124 (53.0%) complete BUSCOs. After several polishing steps with both Nanopore ONT and PB SMRT long reads, it was possible to locate as complete loci 94.7% (2,008) of the BUSCOs on the ONT-based assembly. The use of long read for polishing the ONT-based assembly sequences increased the quality at base level; however, the improvement did not reach the PB-based assembly quality.

Therefore, the 2 assemblies were then combined using RaGOO v.1.1 (Alonge *et al*. 2019), using the higher contiguity of the ONT-based assembly to guide the scaffolding of the higher base-quality PB assembly sequences (Supplementary Fig. 1) and further reduce the number of scaffolds to 236.

### Pseudomolecule creation and gene annotation

To generate the final assembly, we used a very effective approach combining the superior contiguity of the ONT v.1.0 Dovetail assembly and the base quality of the PB Dovetail assembly. RaGOO v.1.1 and MUMmer software v.3.0 software were utilized to generate alignments between these 2 assemblies and then order and orient the PB Dovetail assembly with the ONT v.1.0 Dovetail assembly to produce the final genome assembly—coffee (*C. arabica*) genome UCD v.1.0. The final assembly contains 1,025,606,995 bases, which is 94.5% of the estimated genome size of 1.09 Gb by flow cytometry of the same plant. The assembly has 96.8% of the sequence bases in 22 pseudomolecules corresponding to the 22 chromosomes. The remaining 3.2% of the sequence is in only 214 remaining scaffolds, all >20 kb (Table 1).

BUSCO assessment of the completeness of the final assembly (Table 1) presented a representation of 2,272 out of the 2,326 single-copy orthologs clusters (97.7%) in the eudicotyledons database (odb10). This figure is comparable with the Caturra and Red Bourbon genomes (95.8 and 99.7%, respectively), confirming the high completeness of the assembly and comprehensive representation of the *C. arabica* var. Geisha gene space.

The analysis of repetitive content in the Geisha genome classified 60.6% of the assembly as repetitive. This fraction is consistent with the other *Coffea* genomes (*C. eugenioides* 61.5%, *C. arabica* var. Caturra 62.5%, and *C. arabica* var. Red Bourbon 59.2%), with the exception of *C. canephora* where a figure of 44.8% suggests an incomplete representation of the repetitive content, likely due to technical limitations of the assembly.

RNA-Seq and Iso-Seq data were used as transcriptional experimental evidence for annotation and gene prediction of the Geisha genome. The PB full-length cDNA isoforms were first combined with the mRNA short-read assemblies, first to guide the training of ab initio gene predictors and then to polish the final predictions to obtain alternative splicing events (Supplementary Fig. 2). The annotation of the Geisha genome revealed the presence of 47,062 gene loci encompassing 53,273 protein-coding transcripts (Table 1). Comparing the gene content of the 2 subgenomes, we could identify 8,617 conserved loci that did not encode for a full ORF and have potentially become pseudogenes. The number of loci is consistent for an allotetraploid with the reported numbers from the progenitors’ genomes (25,574 in *C. canephora* and 33,619 in *C. eugenioides*) (Supplementary Table 3).

### Comparison with progenitor species

**Fig. 2.**
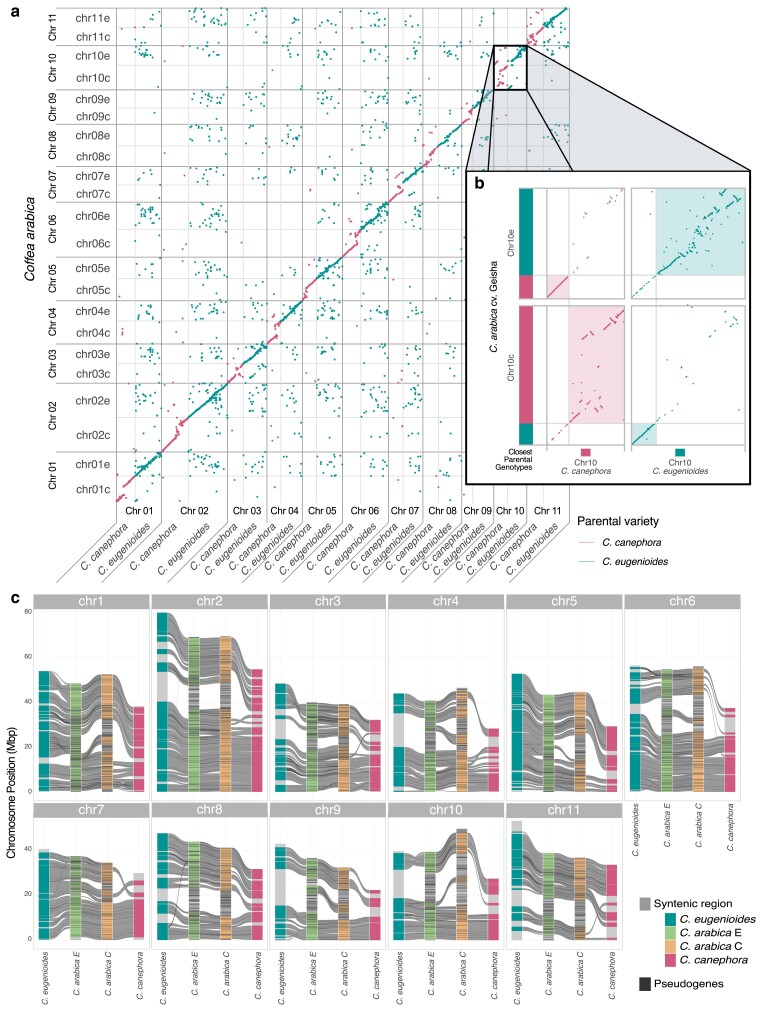
Comparison between *C. arabica*—Geisha and the progenitor species. a) Dot plot diagram comparing the Geisha pseudomolecules to the progenitor chromosome sequences to identify the origin of the subgenomes. Box highlights the location of chromosome 10. b) Expanded view of the alignment of chromosome 10 showing the closest parental genotypes *C. canephora* and *C. eugenioides* for each segment of the pseudomolecules and the switch of inherited content between homeologs. c) Synteny comparison of the chromosome sequences of Geisha and the progenitors species. *C. arabica* E is orthologous to *C. eugenioides* and *C. arabica* C is orthologous to *C. canephora*. Gray connecting ribbons show syntenic blocks of conserved gene loci between pseudomolecules. Black horizontal lines on chromosomes indicate the location of putative noncoding pseudogene loci.

By comparing the pseudomolecules of the UCD v.1.0 assembly to the progenitor genomes available in NCBI, it was possible to separate the set of chromosome sequences inherited from *C. canephora* from the ones inherited from *C. eugenioides* (Fig. 2). Selecting only the alignments with 99% identity between sequences shows a polarization of the hits of each homeolog with just 1 progenitor and the complete loss of alignment across the full length of the pseudochromosome to the sequence of the other progenitor (Fig. 2a and b). As observed for Red Bourbon (Scalabrin *et al*. 2024), for some chromosomes like chromosome 10, we observed a switch of the inherited genetic content between homeologs, with a portion of the pseudomolecule originating from the other progenitor species (Fig. 2b).

The chromosome length, sequence, and structure of the pseudomolecules are closer between the homeologs within *C. arabica*, rather than each subassembly with the original progenitor genome (Fig. 2c). *C. eugenioides* pseudomolecules were overall longer than the associated homeologs in *C. arabica*, confirming what was observed with the flow cytometry that genome size of *C. arabica* is expected to be smaller than the combined length of the progenitors (2.22 vs ∼1.36 + ∼1.46 pg). All *C. canephora* pseudomolecules, on the contrary, show a reduced size when compared with the other assemblies, a situation contradicting the flow cytometry data that would suggest a genome size comparable if not larger than *C. eugenioides*, likely due to a lower assembly quality for the species when compared with the other progenitor.

The comparison of gene content confirms what was observed at the sequence level. Of the annotated loci in the Geisha genome, 89.5% result in syntenic collinear blocks between homeologs. The number drops to 79–82% when comparing each subgenome set with the respective progenitor, for *C. canephora* and *C. eugenioides*. Interestingly, for most of the chromosomes, we can observe a consistency across the intraspecies and interspecies comparisons of which regions appear to be syntenically conserved and regions losing structural coherence (Fig. 2c). Where we observe a loss of synteny between homeologs, those regions seem to accumulate and concentrate pseudogenes (Fig. 2c), suggesting a higher variability or instability leading to the accumulation of deleterious variants for one of the 2 homeolog loci. Also, we observe regions where synteny is maintained only between one of the subassemblies with the respective progenitor, but such a relationship is lost between subassemblies and the other progenitor. This situation is evident on Chr 1 and Chr 5 (Fig. 2c) because of the extent of the synteny of one of the progenitors, but can be observed with different degrees of pervasiveness in all chromosomes.

### Comparison to the Red Bourbon genome sequence

**Fig. 3.**
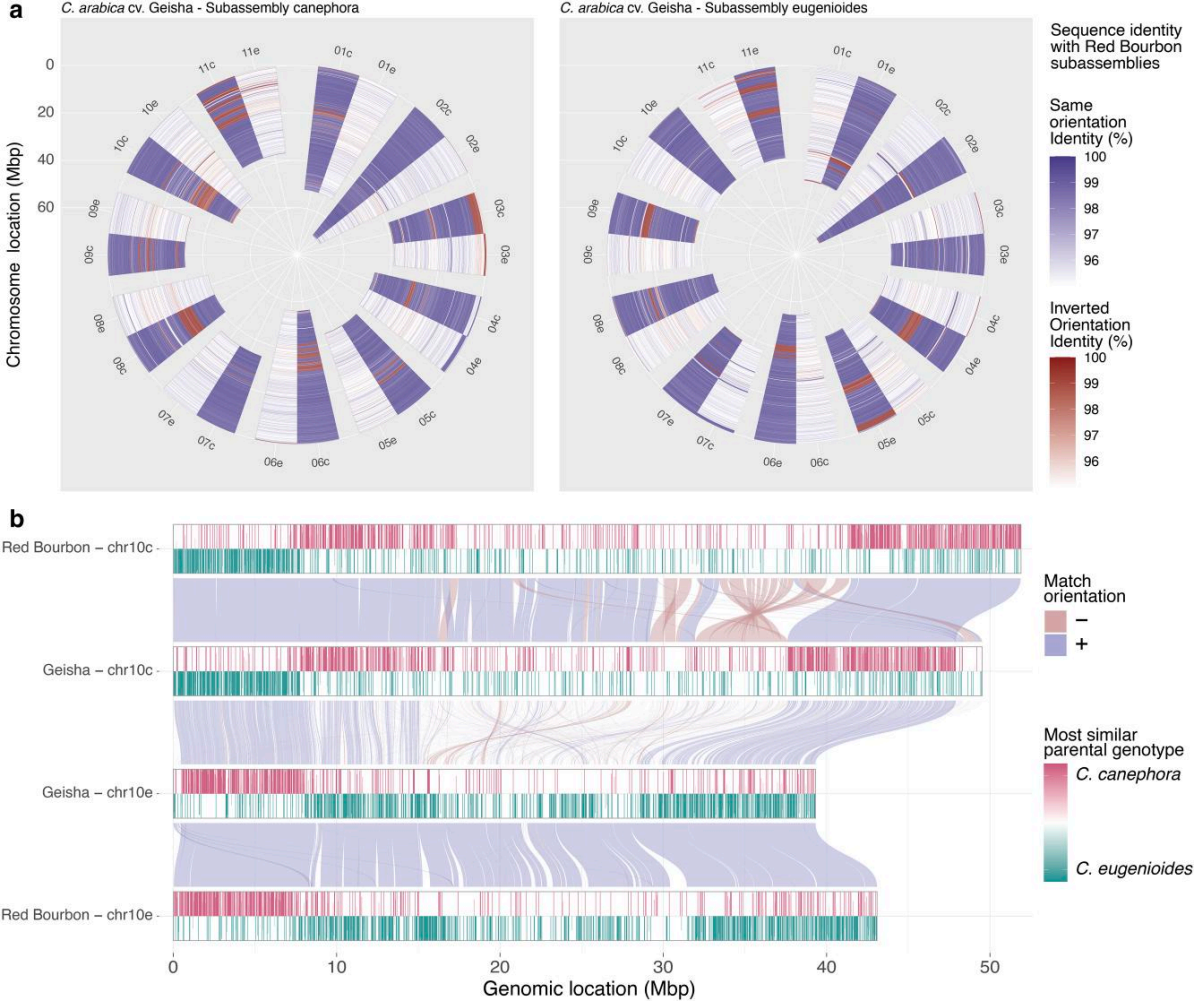
Comparison between the assemblies of *C. arabica* variety Geisha (UCD_1.0) and Red Bourbon (IGA_CARA_2.4). a) Polar diagram of the pseudomolecule identity levels between Geisha and Red Bourbon (blue scale indicates sequence homology in the same orientation; red indicates sequences in inverted orientation). Different levels from the center of the diagram correspond to positions across the length of the chromosomes. b) Alluvial plot showing homolog regions between chromosome 10 pseudomolecules of Geisha and Bourbon varieties. Chromosome bars show color-coded homology levels of pseudomolecule region with the progenitor species (red *C. canephora*; green *C. eugenioides*).

The genomes of Geisha and Red Bourbon varieties were assembled with similar procedures (Scalabrin *et al*. 2024), making them the most comparable from a technical perspective. The structure of all pseudomolecules of both subgenome assemblies C and E is remarkably more similar between the 2 varieties rather than what can be observed when comparing the subgenome assemblies with each of the respective progenitors (Fig. 2a;  Supplementary Figs. 3 and 4). At the nucleotide level, we observe a similar situation. Comparing Geisha and Red Bourbon, corresponding homeologs show a 99.67% median identity (Fig. 3a; Supplementary Fig. 5a), higher than what we observe with the progenitor assemblies (median 97.28%; Supplementary Fig. 5b). Cross-comparing the subgenomes E with subgenomes C shows a homogeneous picture, with a lower median identity of ∼92% regardless if the pair of subgenomes come from the same variety (Supplementary Fig. 5c).

Despite the presence of translocation and inversion events in all homeolog pseudomolecule comparisons, the subassembly E appears to be slightly more conserved in both structure and nucleotide content between varieties and with the progenitor species than their *C. canephora* counterpart (Fig. 2c; Supplementary Fig. 5).

Particularly interesting is the case of chromosome 10, where we observe an exchange of the genetic material inherited from the 2 progenitors between the pseudomolecules. These kinds of conditions are generally ascribed to errors in the assembly procedure, as polyploid genomes can cause the assembly algorithms to switch haplotypes (Chin *et al*. 2016). However, a similar situation has been observed in chromosome 10 pseudomolecules in the independent assembly of Red Bourbon (Scalabrin *et al*. 2024). By comparing the 2 sets of chromosome 10 homeologs (Fig. 3b), we can confirm that both varieties bear a chromosomal exchange event between the 2 progenitor lines. Moreover, the identity in sequence and structure around the switch breakpoints, for both subgenomes of both varieties, suggests that they originated from the very same ancestral recombination event. It is also possible to observe the higher coherence in the structure and the sequence of the regions inherited from C. *eugenioides* than that inherited from *C. canephora*. While they are very similar in sequence content, they show a higher extent of structural rearrangements, and this happens in correspondence of the region that is most dissimilar between the progenitors lines (Fig. 3b). This region appears to be missing or underassembled in the *C. canephora* genome reconstruction (Fig. 2c), although the evolution of the *C. arabica* Chr10c after inheritance from its progenitor cannot be discounted.

It is impossible to trace the evolution of *C. arabica* from the genomic data of just a few reference genomes; however, the distant domestication history between Geisha and Red Bourbon varieties allows us to trace them back to a common origin. The nucleotide identity and the concomitance of the recombination event in chromosome 10 suggest that both varieties derived from the same hybridization event between *C. canephora* and *C. eugenioides*. The evolution of the parental genetic material in chromosome 10 must have happened before the separation of the 2 varieties and the export of the *C. arabica* line that led to the Red Bourbon line from Ethiopia to Yemen to Reunion (Fig. 1). This lends support to Lashermes *et al*. (2016) conclusion of a single allopolyploidization event that gave origin to *C. arabica* after the hybridization event with the 2 progenitor lines and is consistent with Salojärvi *et al*. (2024). Evolution, breeding, and domestication caused the accumulation of the other structural variants we observe that differentiate the structure of the pseudomolecules of Geisha and Red Bourbon. Moreover, the plasticity of the genome appears to be very variable across the different homeologs and different chromosomes.

Comparing different varieties of *C. arabica*, we can assess the quality of genome assemblies and, at the same time, investigate the evolution of the species, impact of domestication events, and allow for precision breeding. The availability of more *Coffea* genomes would also provide a model to understand the mechanisms of allopolyploidization in plants, as it has been observed that when crossing *C. arabica* with a progenitor line hybrid, it can produce an allohexaploid hybrid (Clarindo *et al*. 2013). Currently, few resources are available, as only 2 other *C. arabica* assemblies are accessible (Caturra and Red Bourbon), both resulting from the same line of domestication and distribution (Fig. 1). Therefore, it is important to broaden the availability of high-quality genome assemblies of more *Arabica* varieties, the *Arabica* progenitor lines, and other wild *Coffea* species.

## Supplementary Material

jkae262_Supplementary_Data

## Data Availability

Sequencing data are accessible at the NCBI repository under the accession PRJNA1032043. The Whole Genome Shotgun project has been deposited at DDBJ/ENA/GenBank under the accession JBELAZ000000000. The version described in this paper is version JBELAZ010000000. Final assembly data are available in Zenodo under https://zenodo.org/records/10059814 and in Phytozome, genome ID 871, https://phytozome-next.jgi.doe.gov/info/Carabicageisha_1_0. Pipeline/scripts are available in Supplementary File 1. Supplemental material available at G3 online.
