# Supplementary material for: De novo whole-genome assembly and annotation of Coffea arabica var. Geisha, a high-quality coffee variety from the primary origin of coffee
Source: G3 (Bethesda). 2024 Nov 15;15(1):jkae262. doi: 10.1093/g3journal/jkae262 (PMC11708220; doi:10.1093/g3journal/jkae262)
Supplement: jkae262_Supplementary_Data [file jkae262_supplementary_data.zip › Figure_S3_G3-2024-405138.pdf]

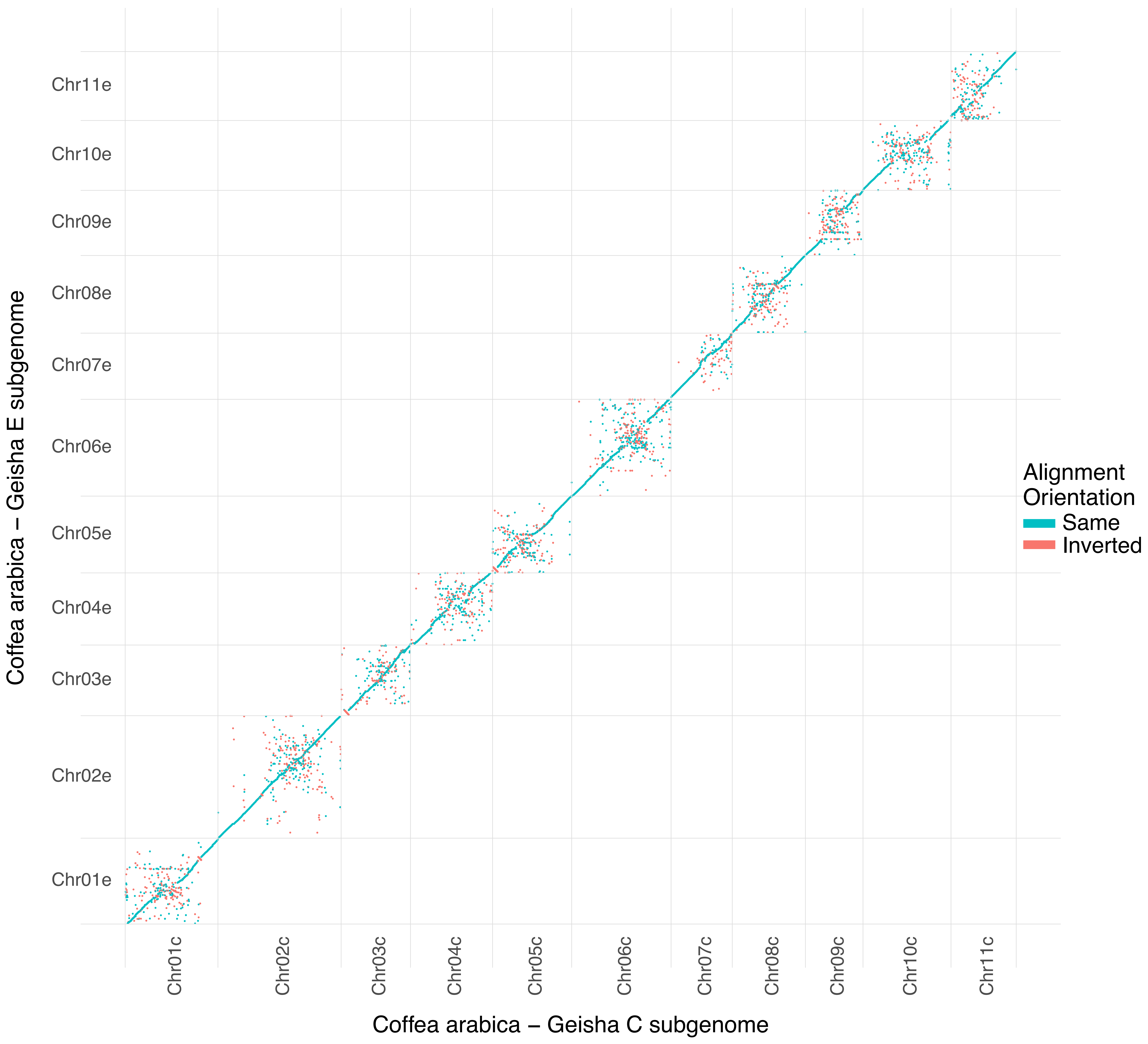
Coffea arabica – Geisha E subgenome
Chr11e
Chr10e
Chr09e
Chr08e
Chr07e
Chr06e
Chr05e
Chr04e
Chr03e
Chr02e
Chr01e
Chr01c
Chr02c
Chr03c
Chr04c
Chr05c
Chr06c
Chr07c
Chr08c
Chr09c
Chr10c
Chr11c
Coffea arabica – Geisha C subgenome
Alignment Orientation
Same
Inverted
