# Supplementary material for: De novo whole-genome assembly and annotation of Coffea arabica var. Geisha, a high-quality coffee variety from the primary origin of coffee
Source: G3 (Bethesda). 2024 Nov 15;15(1):jkae262. doi: 10.1093/g3journal/jkae262 (PMC11708220; doi:10.1093/g3journal/jkae262)
Supplement: jkae262_Supplementary_Data [file jkae262_supplementary_data.zip › File_S1_G3-2024-405138.docx]

Pipeline/scripts for Manuscript G3-2024-405138: De novo whole-genome assembly and annotation of *Coffea arabica* var. Geisha, a high-quality coffee variety from the primary origin of coffee, Medrano et al.

**### ASSEMBLY ###**

# ONT Genome assembly

canu genomeSize=1g minReadLength=5000

# Racon polishing (3 iterations)

## minimap alignment

minimap2 -t 60 -ax map-ont

## polishing

racon (with default parameters)

# Ragoo scaffolding of PacBio assembly with ONT assembly

ragoo -b -t 50 -g 100

**### ANNOTATION ###**

# IsoSeq preprocessing:

ccs --min-rq 0.9

lima --isoseq --dump-clips

isoseq3 refine --require-polya

isoseq3 cluster --use-qvs

#Transcriptome assembly

stringtie -j 2 -c 2

trinity --genome_guided_bam hisat2.bam --genome_guided_max_intron 500000 --long_reads iso-seq.fasta

# Functional annotation

Done with Computomics AnnoScore pipeline

# Gene model filter based on protein similarity and CDS coverage

## Protein alignment to UniProt, SwissProt, Caturra (Diamond database: UniProt Viridiplantae, SwissProt Viridiplantae, 11 November 2020)

diamond blastp --query carabica2_UCD.proteins.fa --db database.db --ultra-sensitive

## Transcript alignment to CDS

bwa mem carabica_CDS.fasta illumina.norm.R1.fasta illumina.norm.R2.fasta

minimap2 -x asm5 carabica_CDS.fasta isoseq_polished.hq.fasta

**### TOOL VERSIONS:**

Canu v1.8

minimap2 v2.17-r941

Racon v1.3.3

RaGOO v1.1

IsoSeq3 v3.3

lima v1.11

Stringtie v2.0

Trinity v2.8.4
