# Supplementary material for: De novo whole-genome assembly and annotation of Coffea arabica var. Geisha, a high-quality coffee variety from the primary origin of coffee
Source: G3 (Bethesda). 2024 Nov 15;15(1):jkae262. doi: 10.1093/g3journal/jkae262 (PMC11708220; doi:10.1093/g3journal/jkae262)
Supplement: jkae262_Supplementary_Data [file jkae262_supplementary_data.zip › Supplemental_Figure_Legends_G3-2024-405138.docx]

**Figure S1: *C. arabica* cv. Geisha UCD v1.0 genome assembly pipeline.** The genome was reconstructed using a novel hybrid *de novo* assembly approach to combine two long-reads single molecule sequencing technologies (Pacific Biosciences SMRT technology and Oxford Nanopore ONT technology), together with Dovetail proximity-ligation sequencing technologies for scaffolding both assemblies. Assembly statistics are shown for the progression of the different steps of the assembly.

**Figure S2: Genome annotation pipeline.** Input for the annotation was a masked version of the genome. RNAseq and Iso-Seq data from 10 different Geisha coffee tissues (Table S1) served as external evidence for gene prediction. Mapped transcriptional data was consolidated to generate a training set for ab-initio predictors. Together with protein evidence from Swiss-Prot and UniProt, consensus gene models were generated using EvidenceModeler and PASA. The final functional annotation integrated data from UniProt and InterProScan databases.

**Figure S3: Dotplot comparing the sequences of the Geisha subgenome assemblies:** Pseudomolecule comparison of the Geisha C subgenome with the Geisha E subgenome highlighting the dissimilarities that exist between the genomes from the respective progenitors. Orientation of the local alignments is color-coded, regions matching in both sequence and orientation are reported in blue while regions matching with inverted orientation are reported in orange.

**Figure S4: Dotplot comparison of the genome assemblies of Geisha and Red Bourbon varieties.** The diagram shows the high similarity that exists between the pseudomolecules of the two varieties. Orientation of the local alignments is color-coded, regions matching in both sequence and orientation are reported in blue while regions matching with inverted orientation are reported in orange.

**Figure S5: Distribution of sequence identity between *Coffea* assemblies.** *C. arabica* Geisha was binned into 25 Kbp windows and compared to the other *Coffea* genome assemblies. Each plot reports, for one pairwise comparison, the unscaled density distribution of the sequence identity measured for each window. **A)** *C. arabica* Geisha subgenomes C and E vs. with *C. Arabica* Red Bourbon subgenomes C and E, **B)** *C. arabica* Geisha subgenomes C and E vs. *C. canephora* and *C. eugenioides* genomes, **C) (left graphs)** *C. arabica* Geisha subgenome C vs *C. arabica* Geisha subgenomes E and *C. arabica* Red Bourbon subgenomes E, **C) (right graphs)** *C. arabica* Geisha subgenome E vs *C. arabica* Geisha subgenomes C and *C. arabica* Red Bourbon subgenomes C.
