# Supplementary material for: De novo whole-genome assembly and annotation of Coffea arabica var. Geisha, a high-quality coffee variety from the primary origin of coffee
Source: G3 (Bethesda). 2024 Nov 15;15(1):jkae262. doi: 10.1093/g3journal/jkae262 (PMC11708220; doi:10.1093/g3journal/jkae262)
Supplement: jkae262_Supplementary_Data [file jkae262_supplementary_data.zip › Table_S1_G3-2024-405138.docx]

| **Table S1: Tissues sequenced for RNASeq and IsoSeq** | | |
| --- | --- | --- |
|  |  |  |
| **Sample** | **RNASeq** | **IsoSeq** |
| Developing berry bean 2mm | X^1^ | - |
| Green bean | X | X |
| Pink bean | X | X |
| Red bean | X | X |
| Pink pulp | X | - |
| Red pulp | X | - |
| Young leaf | X | X |
| Leaf stem | X | - |
| Flower bud 2 mm | X | X |
| Closed flower | - | X |
| Mature flower | X | - |
| Open flower | - | X |
| Root tip 1-2mm | - | X |

^1^ X= sequenced tissues
