# Supplementary material for: De novo whole-genome assembly and annotation of Coffea arabica var. Geisha, a high-quality coffee variety from the primary origin of coffee
Source: G3 (Bethesda). 2024 Nov 15;15(1):jkae262. doi: 10.1093/g3journal/jkae262 (PMC11708220; doi:10.1093/g3journal/jkae262)
Supplement: jkae262_Supplementary_Data [file jkae262_supplementary_data.zip › Table_S2_G3-2024-405138.docx]

| **Table S2: Coffea genome size estimates using Flow Cytometry** | | |  |
| --- | --- | --- | --- |
|  |  |  |  |
| **2C *C. arabica*** | **2C *C. canephora*** | **2C *C. eugenioides*** | **Reference** |
| 2.622 ± 0.002 pg | 1.410 ± 0.002 pg |  | 1 |
| 2.71 ± 0.04 pg | 1.46 ± 0.02 pg |  | 2 |
| 2.47 pg  (2.30-2.72 pg) | 1.46 pg  (1.18-1.61 pg) | 1.36 pg  (1.27-1.43 pg) | 3 |
| 2.61 ± 0.23 pg | 1.54 ± 0.22 pg | 1.39 ± 0.12 pg | 4 |
|  | 1.440 pg (1.423-1.455 pg) | 1.364 pg (1.344-1.384 pg) | 5 |
|  | 1.55 ± 0.08 pg |  | 6 |
| 2.22±0.030 pg |  |  | Geisha UCD1.0 |

**References:**

1 Clarindo WR and Carvalho CR. 2009. Comparison of the *Coffea canephora* and *C. arabica* karyotype based on chromosomal DNA content. Plant Cell Rep. 28:73-81.

2 Clarindo WR, Carvalho CR, Caixeta E T, Koehler AD. 2013. Following the track of ‘Híbrido de Timor’ origin by cytogenetic and flow cytometry approaches. Genet Resour Crop Evol. 60:2253–2259.

3 Cros J, Gavalda MC, Chabrillange N, Recalt C, Duperray C, Hamon S. 1993. Variations in the total nuclear DNA content in African Coffea species (*Rubiaceae*). ASIC, 15th Colloque, Montpellier 1:23-32

4 Cros J, Combes MC, Chabrillange N, Duperray C, Angles AM, Hamon S. 1995. Nuclear DNA content in the subgenus *Coffea* (Rubiaceae): inter- and intra-specific variation in African species. Can J Bot. 73:14–20.

5 Noirot M, Poncet V, Barre P, Hamon P, Hamon S, Kochko A. 2003. Genome size variations in diploid African Coffea species. Ann Bot. 92:709–714.

6 Razafinarivo NJ, Rakotomalala JJ, Brown SC, Bourge M, Hamon S, et al. 2012. Geographical gradients in the genome size variation of wild coffee trees (*Coffea*) native to Africa and Indian Ocean islands. Tree Genetics Genomes 8:1345–1358.
