# Supplementary material for: De novo whole-genome assembly and annotation of Coffea arabica var. Geisha, a high-quality coffee variety from the primary origin of coffee
Source: G3 (Bethesda). 2024 Nov 15;15(1):jkae262. doi: 10.1093/g3journal/jkae262 (PMC11708220; doi:10.1093/g3journal/jkae262)
Supplement: jkae262_Supplementary_Data [file jkae262_supplementary_data.zip › Table_S3_G3-2024-405138.docx]

**Table S3** Genome assemblies and annotation statistics for *C. canephora*, *C. eugenioides* and *C. arabica* – Geisha

| **Assembly** | ***C. canephora*** | ***C. eugenioides*** | ***Geisha -UCD1.0*** |
| --- | --- | --- | --- |
| **Assembly length (Kbp)** | 569,911 | 699,903 | 1,025,607 |
| **Chromosomes** | 11 | 11 | 22 |
| **Unplaced sequences** | 12 | 3,530 | 214 |
| **%GC** | 37 | 37 | 37 |
| **Number of gaps** | 25,532 | 3,482 | 1,876 |
| **Total gap length (Kbp)** | 98,603 | 14,570 | 223.6 |
| **Median scaffold (Kbp)** | 31,808 | 24 | 134.9 |
| **Maximum scaffold (Kbp)** | 205,603 | 79,790 | 69,315.20 |
| **Scaffold N50 (Kbp)** | 38,193 | 47,530 | 43,715 |
| **Scaffold L50 (#)** | 3 | 7 | 10 |
| **Total contigs** | 25,544 | 7,010 | 2,112 |
| **Contigs N50 (Kbp)** | 49.0 | 204.1 | 1107.8 |
| **Contig L50 (#)** | 2,408 | 809 | 234 |
| **Annotation** |  |  |  |
| **Number of gene loci** | 25,574 | 33,619 (1,836 pseudogenes) | 47062 (8,617 pseudogenes) |
| **Number of proteins** | 25,574 | 38,150 | 53,273 |
| **Number of complete BUSCO** | 2,251 | 2,259 | 2,272 |
| **Number of missing BUSCO** | 44 | 46 | 30 |
| **Complete BUSCO (%)** | 96.7 | 97.1 | 97.7 |
| **Repeat (%)** | 44.8 | 61.5 | 60.6 |
