# Supplementary figures and images for: De novo whole-genome assembly and annotation of Coffea arabica var. Geisha, a high-quality coffee variety from the primary origin of coffee
Source: G3 (Bethesda). 2024 Nov 15;15(1):jkae262. doi: 10.1093/g3journal/jkae262 (PMC11708220; doi:10.1093/g3journal/jkae262)

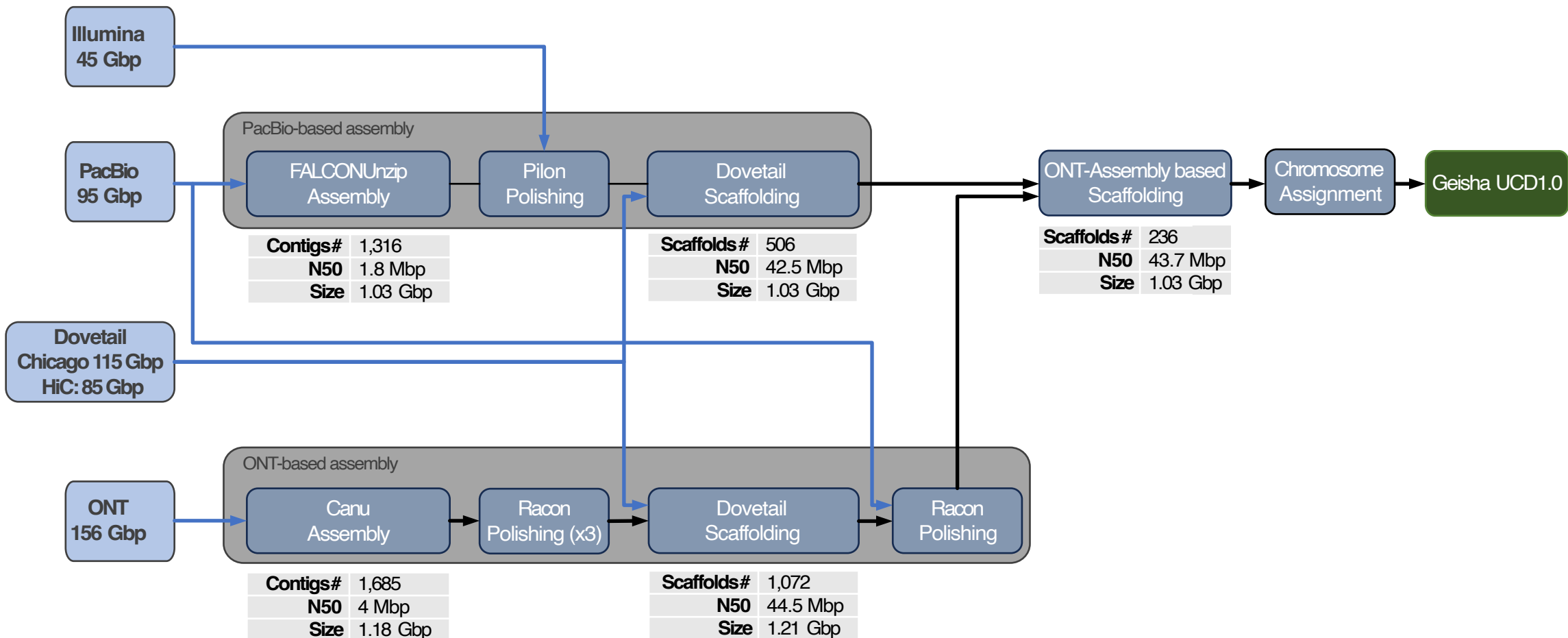

Supplement: jkae262_Supplementary_Data [file jkae262_supplementary_data.zip › Figure_S1_G3-2024-405138.pdf]

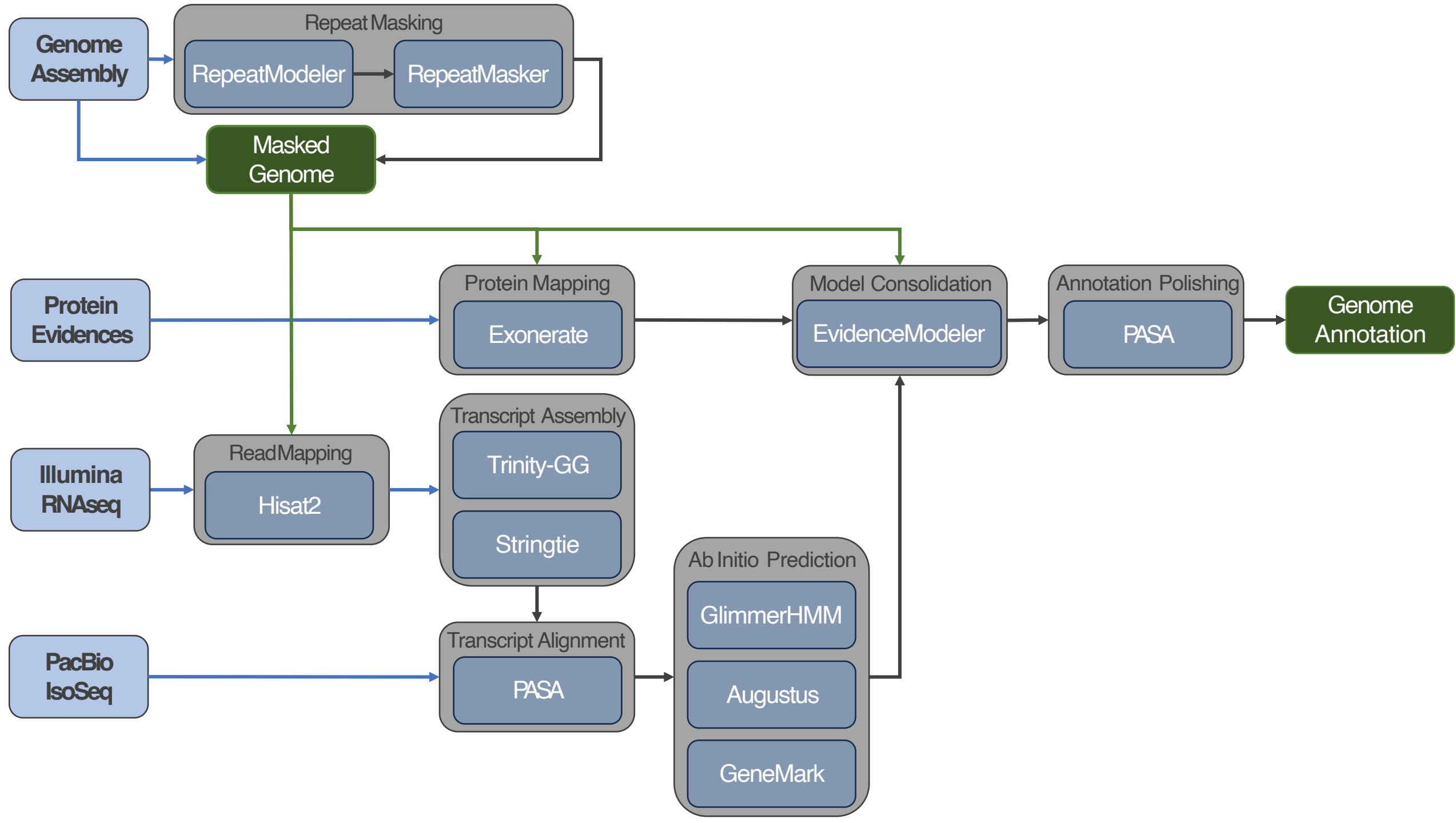

Supplement: jkae262_Supplementary_Data [file jkae262_supplementary_data.zip › Figure_S2_G3-2024-405138.pdf]

Coffea arabica – Red Bourbon

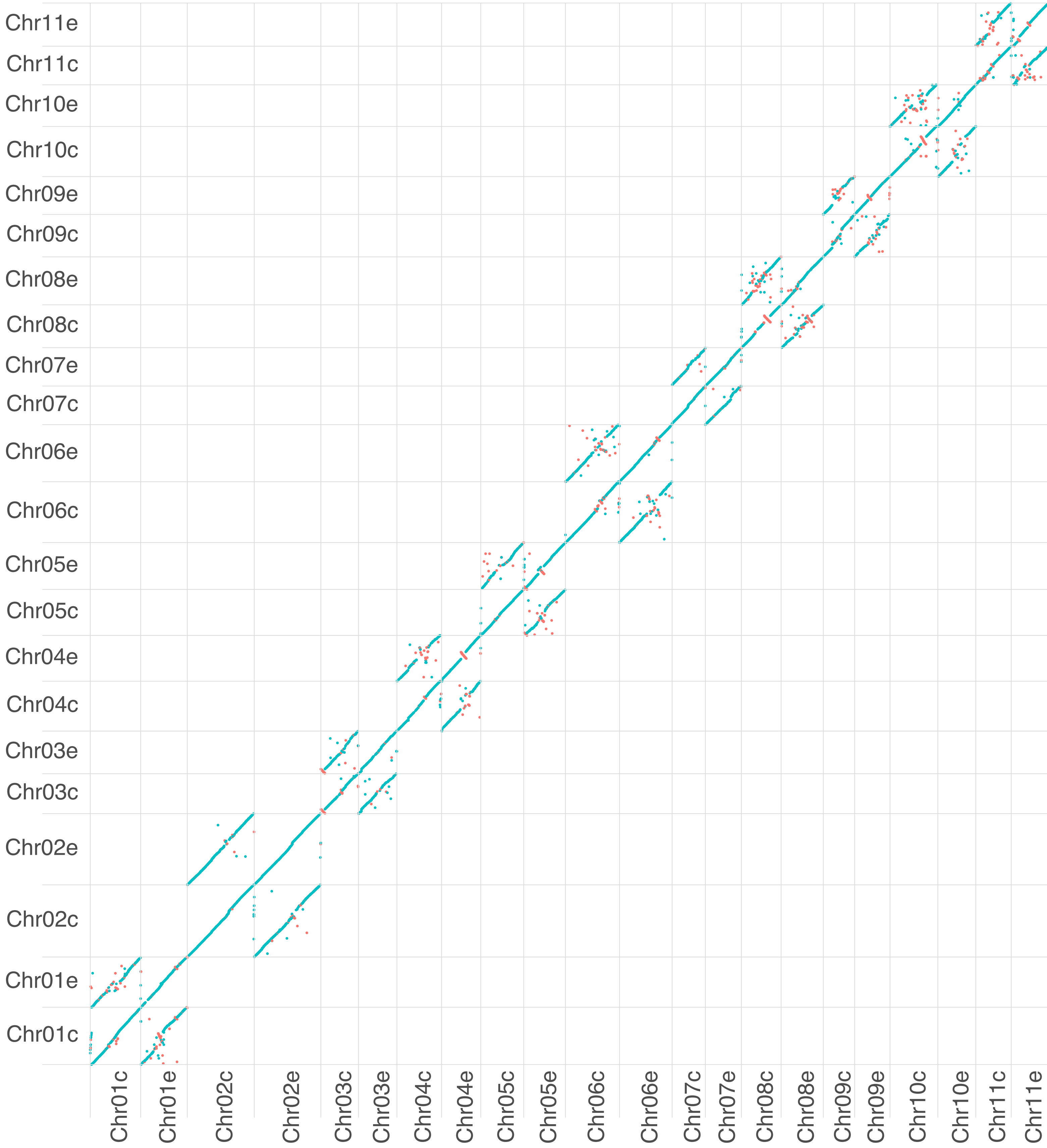

Alignment  
Orientation  
Same  
Inverted

Coffea arabica – Geisha

Supplement: jkae262_Supplementary_Data [file jkae262_supplementary_data.zip › Figure_S4_G3-2024-405138.pdf]

**A**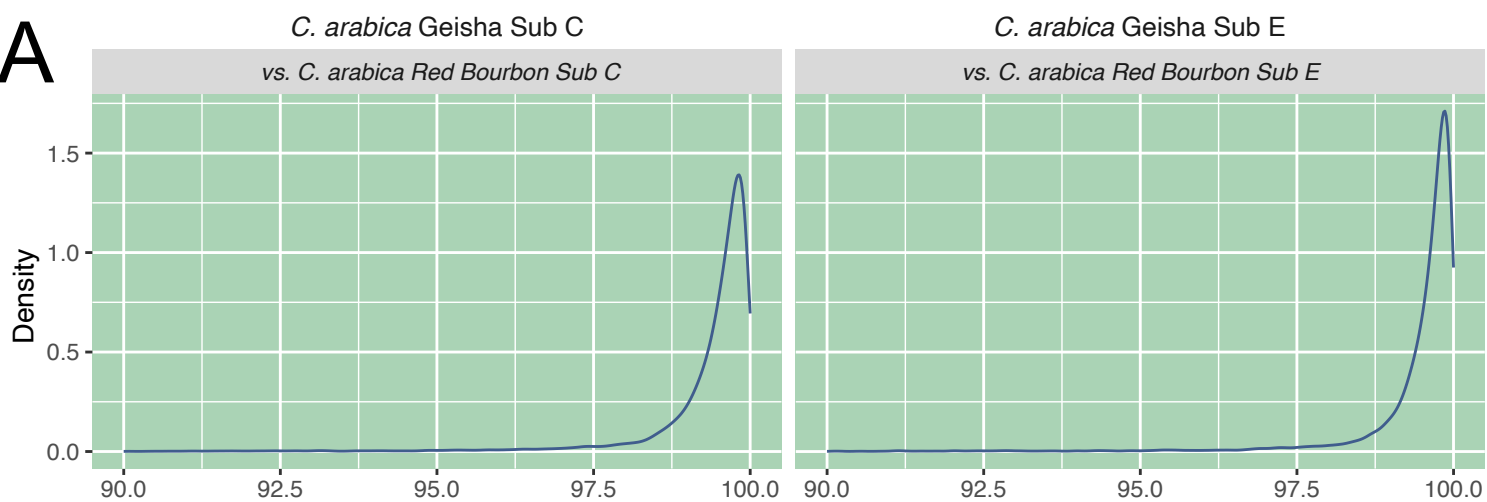**B**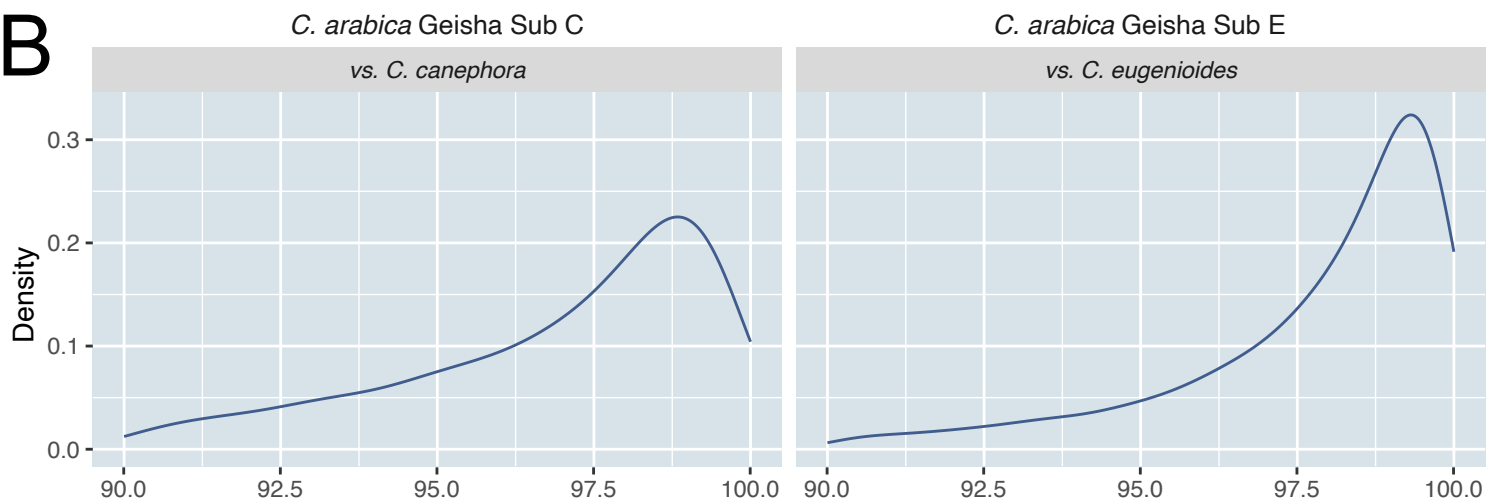**C**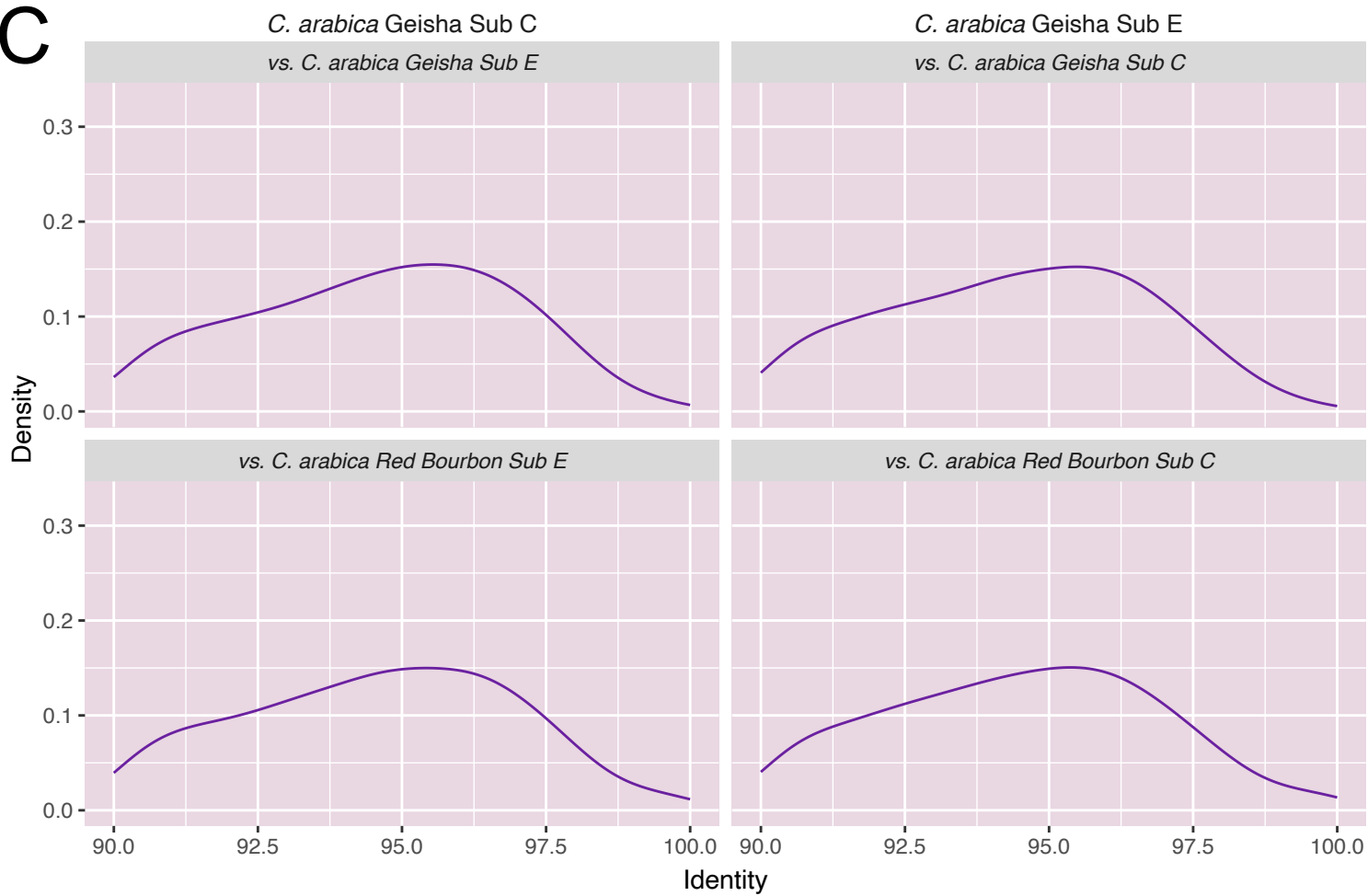

Supplement: jkae262_Supplementary_Data [file jkae262_supplementary_data.zip › Figure_S5_G3-2024-405138.pdf]
